# Supplementary material for: Adhesion G Protein-Coupled Receptor Gpr126 (Adgrg6) Expression Profiling in Diseased Mouse, Rat, and Human Kidneys
Source: Cells. 2024 May 18;13(10):874. doi: 10.3390/cells13100874 (PMC11119830; doi:10.3390/cells13100874)
Supplement: Supplementary file 1 [file cells-13-00874-s001.zip › cells-2982543-supplementary_supplementary figures_R1.pdf]

cells

MDPI

Supplementary information

Adhesion G Protein-Coupled Receptor *Gpr126* (*Adgrg6*) Expression Profiling in Diseased Mouse, Rat, and Human Kidneys

Peter Kösters [1], Salvador Cazorla-Vázquez [1], René Krüger [2], Christoph Daniel [1], Eva Vonbrunn [1], Kerstin Amann [1] and Felix B. Engel [1,*]

[1] Department of Nephropathology, Experimental Renal and Cardiovascular Research, Institute of Pathology, Friedrich-Alexander-Universität Erlangen-Nürnberg (FAU), 91054 Erlangen, Germany; peter.koesters@fau.de (P.K.); s_cazorla@hotmail.com (S.C.-V.); christoph.daniel@uk-erlangen.de (C.D.); eva.vonbrunn@uk-erlangen.de (E.V.); kerstin.amann@uk-erlangen.de (K.A.)
[2] Department of Nephrology and Hypertension, Universitätsklinikum Erlangen, Friedrich-Alexander-Universität Erlangen-Nürnberg (FAU), 91054 Erlangen, Germany; rene.krueger@uk-erlangen.de
* Correspondence: felix.engel@uk-erlangen.de

[1] Department of Nephropathology, Experimental Renal and Cardiovascular Research, Institute of Pathology, Friedrich-Alexander-Universität Erlangen-Nürnberg (FAU), Erlangen, Germany
[2] Department of Nephrology and Hypertension, Universitätsklinikum Erlangen and Friedrich-Alexander-Universität Erlangen-Nürnberg (FAU), Erlangen, Germany
* Correspondence: felix.engel@uk-erlangen.de

Citation: Kösters, P.; Cazorla-Vázquez, S.; Krüger, R.; Daniel, C.; Vonbrunn, E.; Amann, K.; Engel, F.B. Adhesion G Protein-Coupled Receptor *Gpr126* (*Adgrg6*) Expression Profiling in Diseased Mouse, Rat, and Human Kidneys. *Cells* **2024**, *13*, 874. https://doi.org/10.3390/cells13100874

Academic Editor: Jonathon Willets

Received: 10 April 2024
Revised: 10 May 2024
Accepted: 15 May 2024
Published: 18 May 2024

Copyright: © 2024 by the author. Licensee MDPI, Basel, Switzerland. This article is an open access article distributed under the terms and conditions of the Creative Commons Attribution (CC BY) license (https://creativecommons.org/licenses/by/4.0/).

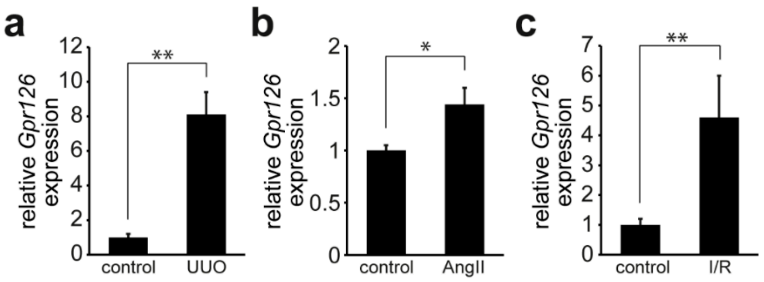

Figure S1. *Gpr126* expression in rodent models of kidney disease. Quantitative RT-PCR analysis on rodent metanephroi in three different models of disease. Graph bars and error bars represent the means ± SD of *Gpr126* relative to *Gapdh* mRNA expression. Statistical analysis was performed using an unpaired t-test. *: p < 0.05, **: p < 0.01 (a) Mouse model of unilateral ureteral obstruction (UUO); n = 5 (control) and 6 (model) independent experiments. (b) Mouse model of angiotensin II perfusion (AngII); n = 4 (control) and 6 (model) independent experiments. (c) Rat model of ischemia-reperfusion injury (I/R); n = 6 independent experiments.

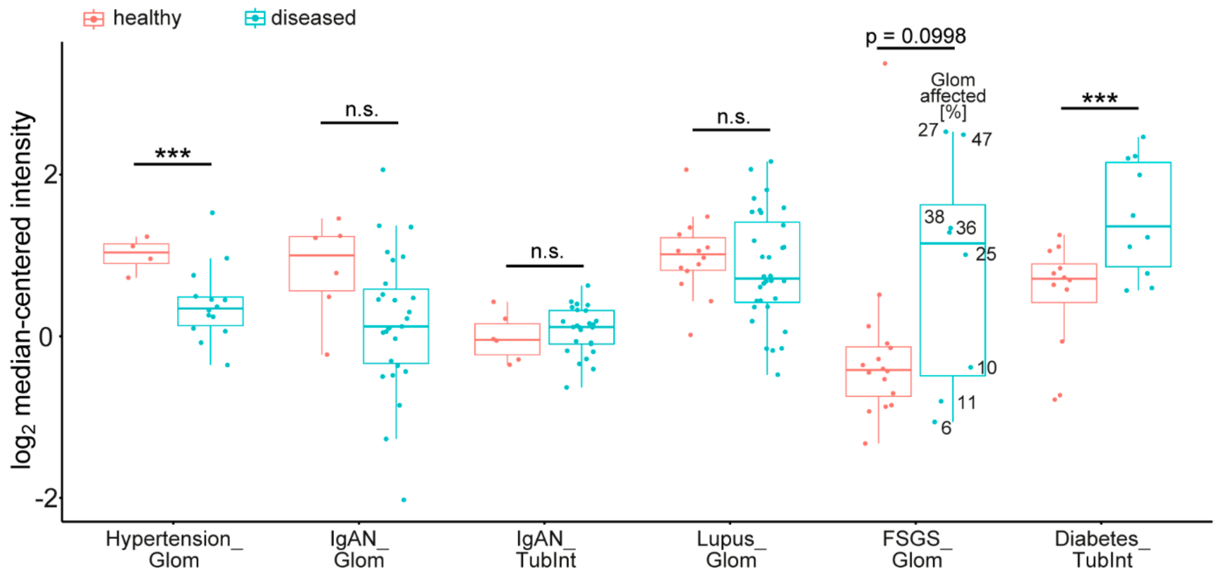

Figure S2. Analysis of changes in Gpr126 expression in human kidney disease based on the NephroSeq data. To compare *Gpr126* expression in healthy living donors to that in individuals with kidney disease, we conducted a statistical analysis using a two-sided t-test via rstatix and visualized log2 median-centered intensity with ggpubr https://cran.r-project.org/web/packages/ggpubr/index.html. Lupus_Glom (Lupus nephritis; glomeruli; control: n = 14; diseases: n = 32), IgAN_TubInt (IgA nephropathy; tubulointerstitium ;control: n = 6; diseases: n = 25), IgAN_Glom (IgA nephropathy; glomeruli; control: n = 6; diseases: n = 27), FSGS_Glom (FSGS, glomeruli; control: n = 16; diseases: n = 8), Hypertension_Glom (hypertension; glomeruli; control: n = 4; diseases: n = 14), and Diabetes_TubInt (diabetes; tubulointerstitium; control: n = 10; diseases: n = 12). ***: p < 0.005. n.s.: not significant.

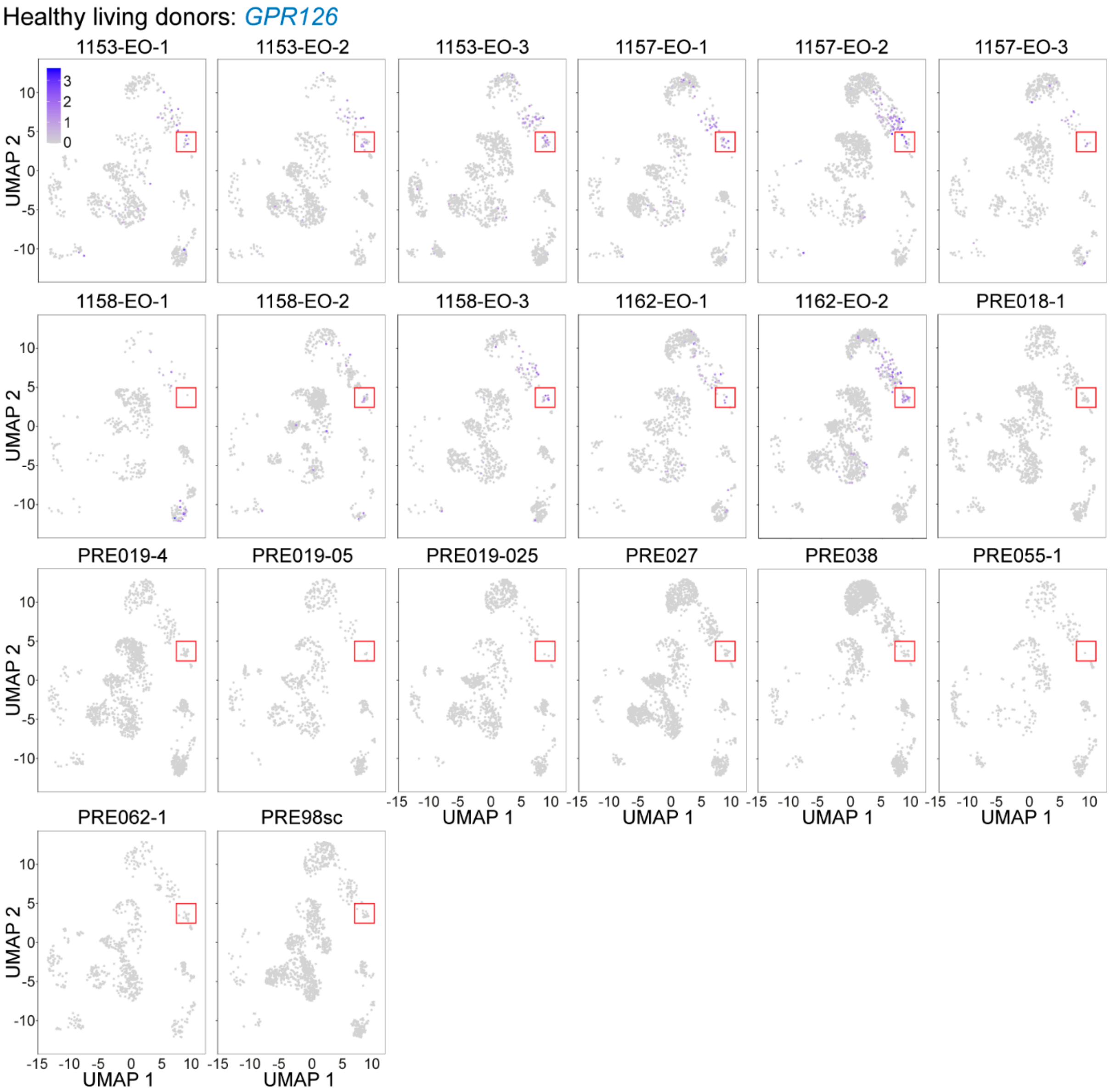

Figure S3. Individual UMAP plots of *GPR126* expression for each of the 20 healthy living donors (control).

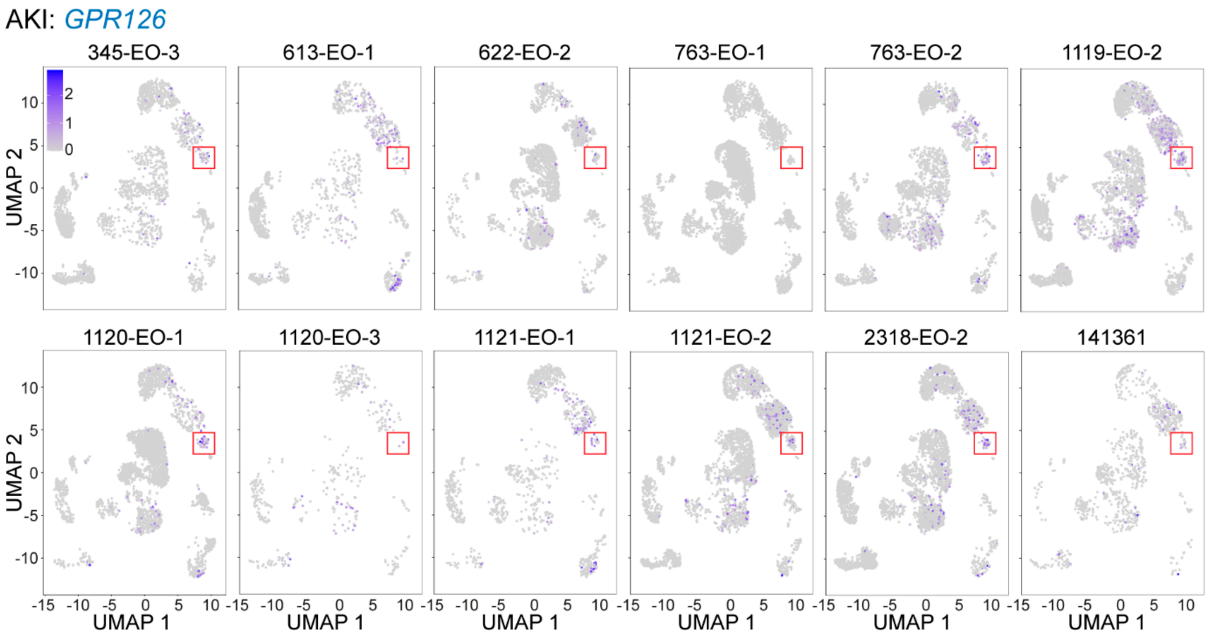

Figure S4. Individual UMAP plots of *GPR126* expression for each oft he 12 AKI patients.

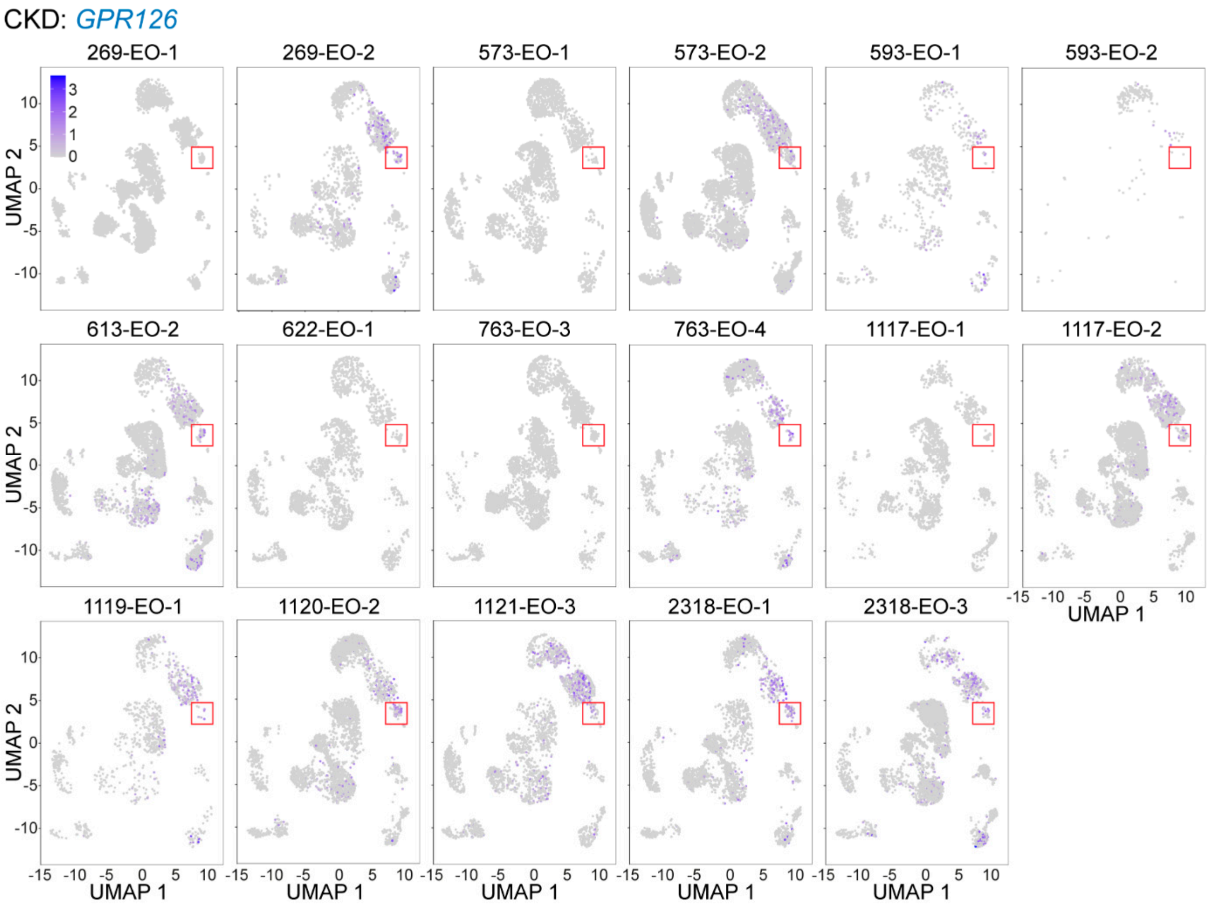

Figure S5. Individual UMAP plots of *GPR126* expression for each oft he 17 CKD patients.

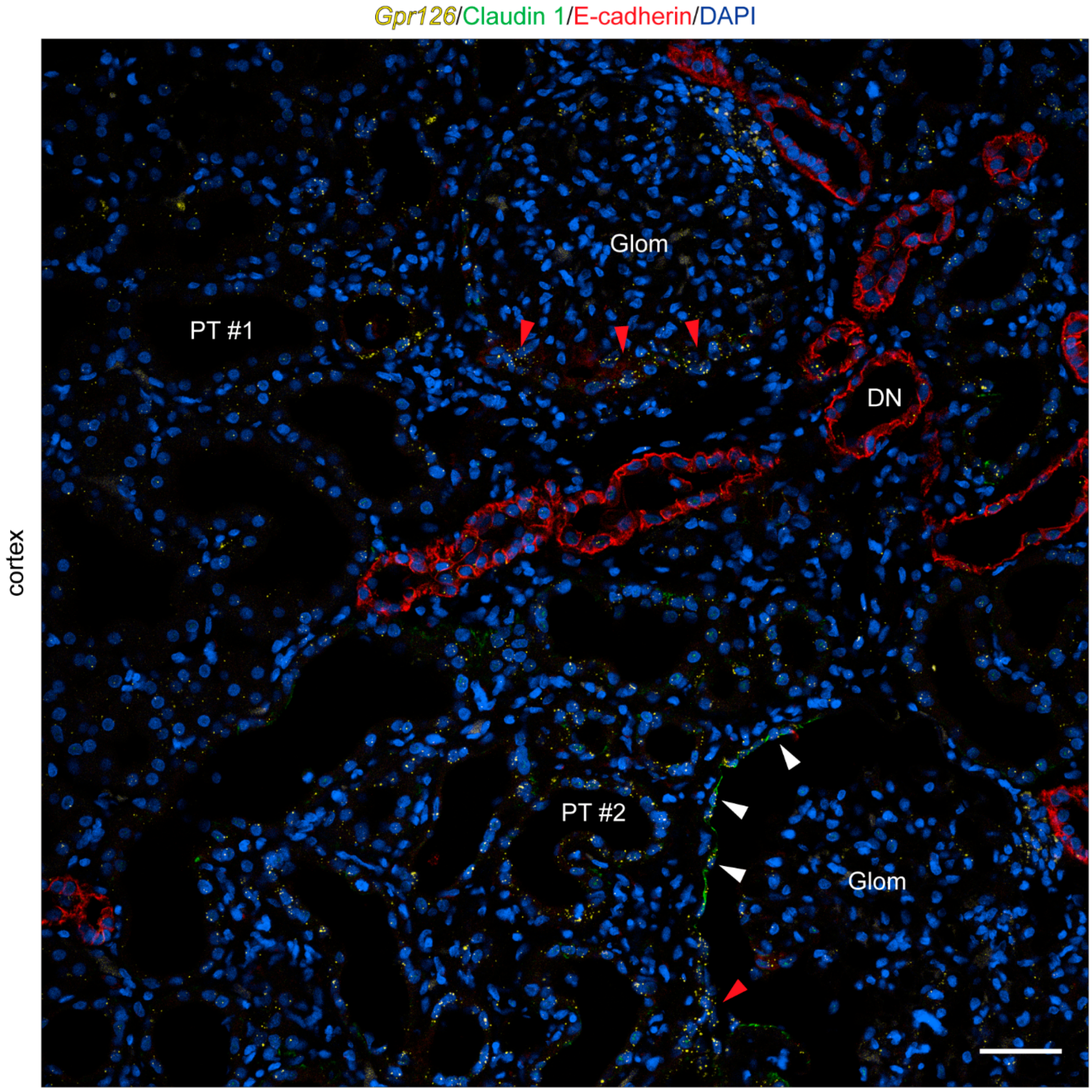

Figure S6. Overview RNAscope® in situ hybridization image of kidney cortex from FSGS patient biopsy. Proximal tubules (PT, Claudin 1-negative, E-cadherin-negative) show different levels of GPR126 expression (yellow dots; PT #1: low expression; PT #2: high expression). Parietal epithelial cells (PECs) inside FSGS lesions (red arrowheads, Claudin 1-negative) show increased expression of GPR126 compared to PECs outside FSGS lesions (white arrowheads, Claudin 1-positive). There is no increase of GPR126 expression in the distal nephron segments (DN, E-cadherin-positive). Scale bar: 50 μm.

Disclaimer/Publisher's Note: The statements, opinions and data contained in all publications are solely those of the individual author(s) and contributor(s) and not of MDPI and/or the editor(s). MDPI and/or the editor(s) disclaim responsibility for any injury to people or property resulting from any ideas, methods, instructions or products referred to in the content.
